# Putting patients first: development of a patient advocate and general practitioner-informed model of patient-centred care

**DOI:** 10.1186/s12913-021-06273-y

**Published:** 2021-03-20

**Authors:** Bryce Brickley, Lauren T. Williams, Mark Morgan, Alyson Ross, Kellie Trigger, Lauren Ball

**Affiliations:** 1 Menzies Health Institute Queensland, Griffith University, Gold Coast, QLD Australia; 2 Bond University, Gold Coast, QLD Australia; 3 Gold Coast Primary Health Network, Gold Coast, QLD Australia

**Keywords:** Patient-centred care, General practitioners, Primary health care, General practice, Quality, Safety, Qualitative description

## Abstract

**Background:**

Patients, providers and health care organisations benefit from an increased understanding and implementation of patient-centred care (PCC) by general practitioners (GPs). This study aimed to evaluate and advance a theoretical model of PCC developed in consultation with practising GPs and patient advocates.

**Methods:**

Qualitative description in a social constructivist/interpretivist paradigm. Participants were purposively sampled from six primary care organisations in south east Queensland/northern New South Wales, Australia. Participants engaged in focus group discussions where they expressed their perceptions, views and feelings of an existing PCC model. Data was analysed thematically using a constant-comparison approach.

**Results:**

Three focus groups with 15 patient advocates and three focus groups with 12 GPs were conducted before thematic saturation was obtained. Three themes emerged: i) the model represents the ideal, ii) considering the system and collaborating in care and iii) optimising the general practice environment. The themes related to participants’ impression of the model and new components of PCC perceived to be experienced in the ‘real world’. The data was synthesised to produce an advanced model of PCC named, “*Putting Patients First: A Map for PCC*”.

**Conclusions:**

Our revised PCC model represents an enhanced understanding of PCC in the ‘real world’ and can be used to inform patients, providers and health organisations striving for PCC. Qualitative testing advanced and supported the credibility of the model and expanded its application beyond the doctor-patient encounter. Future work could incorporate our map for PCC in tool/tool kits designed to support GPs and general practice with PCC.

**Supplementary Information:**

The online version contains supplementary material available at 10.1186/s12913-021-06273-y.

## Background

Patient-centred care (PCC) is care that is respectful and responsive to the wishes of patients [1]. In 2001, the U.S. based National Academy of Medicine (formerly, Institute of Medicine) nominated PCC a key objective for improving health care in the twenty-first century [2]. High levels of PCC have been associated with improved health outcomes [3–6], enhanced relationships between providers and patients [5], enhanced patient satisfaction [3, 7] and greater adherence to treatment [4]. Clearly, PCC is valuable to patients, providers and health care organizations.

Family physicians, also called general practitioners (GPs), are well-positioned to provide PCC because they are usually the first contact for patients entering health systems [8, 9]. Practising GPs need to be up-to-date consumers of research including PCC, to deliver high-quality and low-risk care [9]. Research on PCC has previously been synthesised through reviews and concept analyses, resulting in conceptual models that can be utilised by health care providers [10–12]. Existing models of PCC vary in their relevance to a specific health setting or provider [10, 11, 13–15].

Models of PCC are essential to supporting the understanding of PCC and the extent to which it is achieved in practice. One of the most commonly applied PCC models was published in 2014 by Scholl and colleagues, who reviewed 417 articles situated in primary, tertiary and acute care. This PCC model consists of 15 distinct dimensions, but its applicability to GPs and general practices is limited due to limited included studies being situated in primary care (< 5%) [10]. The most recently published model of PCC for GPs was published in 2019 by Brickley and colleagues [14]. This model is highly theoretical because it arose from an integrative review of predominantly quantitative articles but also, systematic review, mixed methods and only three qualitative articles [14]. Furthermore, included studies needed to refer to both a GP and patient and consequently, most studies took place in the micro-level of health care (doctor-patient encounter) [14]. The Brickley and colleagues’ model [14] places greater emphasis on the micro-level of health care compared to the practice-level. The model is yet to be evaluated and its ability to inform the development of an implementation tool kit is unknown.

In the ‘real world’, PCC is derived from the context to which it is implemented, social experience and individual perspectives [16]. These factors can only be captured by qualitative study design [14, 17, 18]. Patient advocates offer unique perspectives because they are typically trained and experienced in research, and they consider the significance for patients throughout the research process [19]. Furthermore, they tend to offer an ‘expert’ perspective on PCC because of their likely deeper understanding of the health system compared with typical patients. Consultation with patient advocates and GPs can inform new ideas for supporting PCC.

This study aimed to evaluate the recent model of PCC for GPs developed by Brickley and colleagues [14], in collaboration with GPs and patient advocates, to advance a model of PCC. A PCC model-informed implementation tool kit has the potential to be valuable to policy makers, patients, health professionals, health systems and researchers to inform PCC research.

## Methods

### Study design

**Fig. 1:**
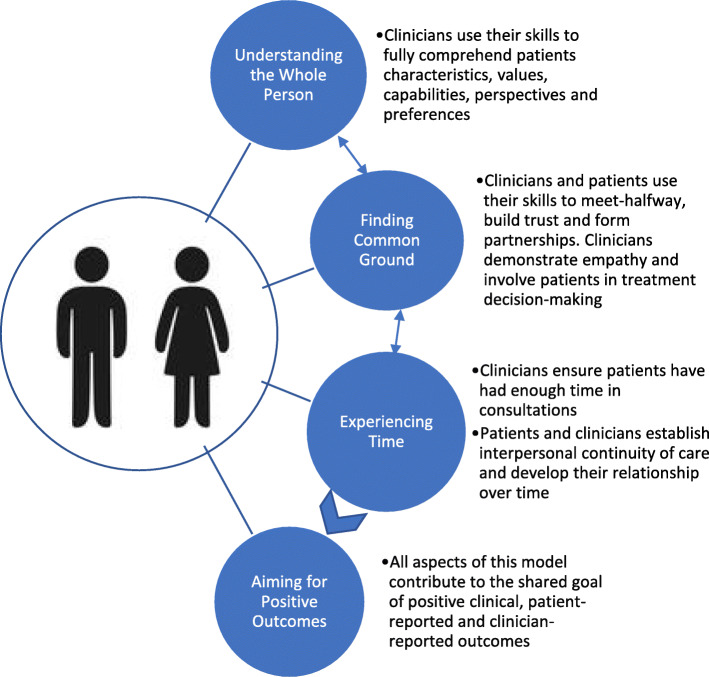
Model of Patient-Centred Care for General Practitioners [14]

This study was conducted from a social constructivist/interpretive philosophical position [20]. The research question was “How do patients and GPs perceive the model of PCC for GPs, published by Brickley and colleagues in 2019 [14]?” This model is comprised of four inter-related components and is displayed in Fig. 1. To address the research question, this study employed a qualitative descriptive approach, utilising focus groups with patient advocates and focus groups with GPs. Focus groups commenced with a broad exploration of GP-delivered PCC, which has been published elsewhere [21]. Ethics approval was obtained from the Griffith University Human Research Ethics Committee (No. 2019/634).

### Selection and recruitment of study population

Patient advocate and GP participants were recruited from six primary care organisations in south east Queensland and northern New South Wales (NSW), Australia. Three patient advocacy group managers and three general practice managers who were known to researchers were purposely engaged via email to assist in the recruitment of participants. Study information sheets were provided to managers and recruitment snowballed through their respective organisations. Patient advocates are those who participate in health consumer engagement, advisory or consultant groups and ‘advocate’ on the behalf of others [22]. The involvement of patient advocates in research has been shown to produce health information that meets the needs of consumers, assist with bridging the gap between research and practice [23], and increase the quality of services [24]. Eligible patient advocates were English speaking adults, who had participated in at least one recent GP consultation (< 3 months) and were currently participating in patient engagement activities. We considered engagement activities to be any formal role where an individual is advocating in health care on behalf of other patients. Eligible GP participants were English speaking adults who were currently practising.

### Interview protocol

Focus groups contained structured questions outlined in an interview guide developed by the research team (Table 1). The interview guide was informed by Kvale’s (1996) stages of conducting in-depth interviews [25], then tailored for use in a focus group. The interview guide was piloted with four purposely sampled patients, and then modified prior to data collection. The facilitator (BB) initially provided participants with a hard copy of Brickley and colleagues’ PCC model [14]. Participants were given time to read the model, provided with an opportunity to ask questions and clarify their understanding, and were encouraged to reflect on their ‘real world’ experiences of PCC in consideration of the model. The facilitator then posed questions to the group in accordance with the structured guide. To advance conceptual thinking, probing questions were added to encourage participants to elaborate on initial ideas. All focus groups were ~ 30 min in duration. After the first focus group and initial analysis, a theoretical sampling technique was used to revise and adapt the interview process as the research progressed [26]. Interviews were redirected to focus on new concepts as some components of PCC became saturated with explanatory data. This was an iterative process and researchers explored emerging codes and themes in subsequent focus groups.

**Table 1:** Focus Group Interview Guide

Research Question	Inquiry Purpose	Main Interview Questions (GPs)	Main Interview Questions (Patients)	Potential Probing Questions
What are patients and GPs perceptions of the theoretical model of PCC (identified in phase 1)?	1. Provide opportunity for open expression of views regarding the theoretical model of PCC. 2. Determine the perceived similarities and differences of the theoretical model of PCC to participants lived experiences of PCC. 3. Identify experiences that describe the important elements of ‘real world’ PCC.	1. Can you elaborate on your views and feelings of the model of PCC? 2. Have you felt that care you have provided in the past included all aspects of this model? 3. Can you give a more detailed description about what this model lacks in respect to ‘real world’ application of PCC?	1. Can you elaborate on your experience, views and feelings of the model of PCC discussed in the briefing? 2. Do you feel that you have experienced all aspects of this model in care from your GP? 3. Can you give a more detailed description about what this model lacks in respect to real ‘world’ application of PCC?	1. Are there any other aspects of your experience that is important to you? 2. Do you feel as if your peers feel the same way about this as you?

Abbreviations: *PCC* Patient-centred care, *GP* General practitioner

### Data collection

Participants’ postcodes of residence were recorded. All other data collection and analysis were completed simultaneously and the sample size were determined when thematic saturation was reached [27]. An iterative approach of purposive sampling was undertaken to ensure data saturation [27]. Focus groups were audio-recorded using a dictaphone and subsequently transcribed for analysis. One of the three patient advocate focus groups was moderated by a researcher (KT) who is a patient advocacy group manager. One of the three GP focus groups was moderated by a researcher (MM) who is a GP. The primary researcher (BB), who is a PhD Candidate, served as both facilitator and moderator in the remaining four focus groups. Moderators used their background and skills to maintain a controlled, open dialogue in the focus group; to add scrutiny to concepts that arose and to make detailed notes, which assisted with analysis. Participants were invited to verify the accuracy of the transcript with their contribution to the focus group.

### Data analysis

Participants’ geographic information was interpreted using scores from the accessibility/remoteness index of Australia (ARIA) [28]. Qualitative data was analysed using a constant comparison method and the six phases of thematic analysis [29]. Data analysis commenced simultaneously with data collection, where researchers generated initial ideas of themes to explore in subsequent interviews. Field notes supported the analytical process. No further focus groups were required when thematic saturation was obtained; which was identified by research team consensus as analysis failed to yield any new codes or themes [27]. The analytical process was highly reflective, and the entire research team took part in reviewing, defining and naming themes [17].

### Model development

The primary researcher (BB) reflected on the present study’s interview data and its relationship to Brickley and colleagues’ existing PCC model [14]. The entire research team then revised the conceptual model, to be inclusive of as much data as possible. New elements of PCC were synthesised with the existing model to form an advanced, integrated summation of PCC for GPs and general practices. The components and design of the advanced model were continually discussed by the research team until a consensus was reached. The primary researcher (BB) collaborated with a local graphic design company to produce a visually appealing map for PCC that was purposely designed for use by patients, GPs and general practices.

### Model verification

Once the model was developed, one practice manager and one advocacy group manager were approached to refer participants for an additional verification step. The newly developed model of PCC was presented to a group of patient advocates during a local consumer advisory council meeting, some, but not all the 15 patient advocate attendees had participated in focus group one of this study. The model of PCC was also presented to three GPs, one of whom had participated in focus group four, whilst the other two were colleagues of the previous participant. Participants were invited to verify the accuracy of the advanced model with an emphasis on its applicability to the ‘real world’. This stage informed further development on the model to ensure it closely reflected participants ideas and was inclusive as much data as possible.

## Results

### Participants

Twenty-seven participants were involved in focus groups between September 2019 and November 2019. Participants’ individual characteristics are displayed in Table 2. There were 15 patient advocates (5 male, mean age (SD) 57 [19] years) and 12 GPs (6 male, mean age (SD) 53 [12] years). Nearly all participants (93%) resided in major cities with relatively unrestricted access to goods and services [28].

**Table 2:** Individual Characteristics

Focus Group (*n*)	PA	Gender (M/F)	Age (y)	ARIA classification	Duration (mins)
1 (moderated by KT)	1	F	22	MC	83.0
	2	M	20	MC	
	3	F	47	MC	
	4	M	68	MC	
	5	F	61	MC	
	6	F	69	MC	
2	7	F	38	MC	50.0
	8	F	75	MC	
3	9	F	80	MC	69.0
	10	M	81	MC	
	11	F	44	MC	
	12	F	68	MC	
	13	F	59	OR	
	14	M	64	MC	
	15	M	61	OR	
**Focus Group (*n*)**	**GP**	**Sex (M/F)**	**Age (y)**	**ARIA classification**	**Duration (mins)**
4	1	F	43	MC	30.0
	2	M	70	MC	
	3	M	65	MC	
5 (Moderated by MM)	4	F	55	MC	33.0
	5	F	40	MC	
	6	M	67	MC	
	7	M	62	MC	
	8	M	43	MC	
	9	M	39	MC	
	10	M	63	MC	
6	11	F	43	MC	37.0
	12	F	47	MC	

Abbreviations: *ARIA* Accessibility Remoteness Index of Australia, *GP* General practitioner, *MC* major city, *OR* outer regional, *PA* patient advocate

### Thematic analysis

Three themes emerged from our analysis that related to participants’ overall impression on the model and gaps that were identified: i) model represents the ideal of PCC, ii) considering the system and collaborating in care and iii) optimising the general practice environment. We also noted general suggestions from participants to improve the model’s application to the ‘real world’ (Supplementary Table 1). These did not emerge as main themes as they were mentioned in brief but were important to the advancement of the PCC model. Interpretive findings are described below and supported with narrative quotes. Patient advocate data is indicated by PA1–15 and GP data by GP1–12.

#### Model represents the ideal of patient-centred care

Participants’ ideals of PCC aligned with the components displayed in the model. Providers’ said, “it’s all ideal” (GP2); “I agree with all of it, it’s got everything to it. It’s what I thought it [PCC] was” (GP9). Patient advocates said, “this is a perfect model” (PA3); “In a perfect world, every GP would follow this model” (PA1). However, there was uncertainty to whether it is possible to achieve all components of this model universally in practice. One patient advocate said they had never experienced PCC consistent with the model; “If you showed me a doctor that does this I would go to that doctor” (PA3). Some providers aimed to implement all aspects of the model but indicated that it is not always achievable, “I’m sure this is what most of us would be aiming for” (GP2). One GP suggested that the wording of the model was particularly idealistic, and that it should be changed to be more realistic, “clinicians try to ensure patients have had enough time in consultations” (GP3).

#### Considering the system and collaborating in care

The complexity of the local health system was reported by both GPs and patient advocates to be a common barrier to PCC. One GP felt a barrier to PCC to be “trying to understand the public system” (GP2). A patient advocate expressed “I’ve had to find [services] myself because doctors didn’t have that knowledge, and because it wasn’t easy to find” (PA7). One GP reflected on an experience regarding care coordination, which was made difficult due to a complex health system, “it could be a year’s wait before he gets reviewed … you’ve got to get him seen faster than that” (GP3).

Cost and remuneration were key considerations of GPs when striving for PCC. One GP felt that the current health system’s funding arrangements caused personal financial pressures for GPs, which led them to compromise PCC. Similarly, one other GP reported to act against the wishes of a patient if the cost to the health system is too high:

I may refuse to do an MRI [magnetic resonance imaging] if they [patients] insist on it, if I don’t think it’s appropriate, and I think cost is important, we are the gatekeepers (GP6).

Time poor general practice business models, such as “turnstile type medical practices” (GP3); and policy “Medicare is underfunded without doubt for general practice” (GP6) exacerbated time and financial pressure placed on GPs. One solution for patients and GPs to mitigate the complexity of the health system while striving for PCC was to collaborate with other health professionals, peers, and organisations. One GP stated,

It’s very important that we can connect [patients] to helpful resources and allied health professionals. We cannot do everything in one sitting, we are just one person (GP12).

#### Optimising the general practice environment

Patient advocates viewed the entire general practice environment as an important influence on the extent to which PCC is achieved; “person-centred care starts as you walk in the door!” (PA11). Patient advocates associated the environment with outcomes; “the environment will facilitate the opportunity to have a successful outcome” (PA14). In this context, general practice reception staff were regarded as having a role in PCC because they can “help someone feel at ease … communication, respect and safety start with reception” (PA12). Patient advocates noted that environmental design (e.g. purposeful equipment placement, colours and sounds), general practice culture and reception staff had the potential to promote PCC. One patient advocate (PA14) recounted the experience of a service ‘walk through’. The participant described how valuable his feedback was to the patient-centredness of the service:

… someone painted it, changed the seats, changed the whole format, gave them a little bit of [further] advice, and the next time I went it I was like wow! You could feel the [patient-centred] culture from the moment you got there (PA14).

### Conceptualisation and verification of putting patients first: a map for PCC

**Fig. 2:**
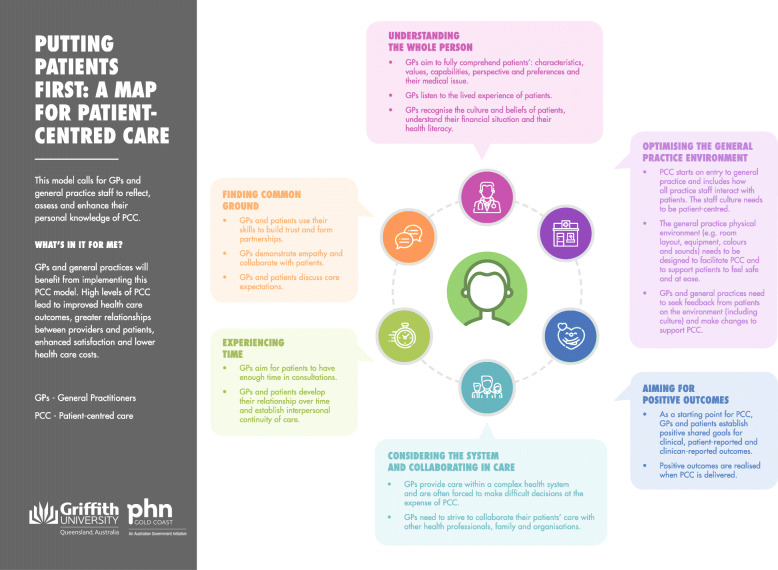
Putting Patients First: A Map for Patient-Centred Care (developed by our research team)

Putting patients first:*A Map for PCC* was conceptualised from the data and is displayed in Fig. 2. The map illustrates the integration of new PCC components: considering the system and collaborating in care; and optimising the general practice environment. The verification groups called for the text in the map to make explicit the incentive to deliver PCC, and to be orientated to engage general practice managers, all general practice staff and patients. A purpose statement and ‘what’s in it for me’ statement were added and the six components of PCC were supported with text descriptors as a guide for key stakeholders of PCC in general practice.

## Discussion

Putting patients first:*A Map for PCC* was synthesised from the evaluation of an existing model of PCC for GPs [14] in consultation with GPs and patient advocates. This study supported the Map for PCCs readiness for practice, as qualitative testing has contextualised the model to the ‘real world’ and extended its application to beyond the micro-level of health care. Our model provides an enhanced conceptual understanding of PCC that can be used to support the development of tools/tool kits and future research promoting the adoption and implementation of PCC.

An evaluation of the U.K. based MAGIC programme found that a lack of support tools and the day-to-day demands and priorities for GPs inhibited shared decision-making, which is a fundamental component of PCC [30]. These findings are reflected in our study as both patient advocates and GPs identified that GPs need support for PCC. Providers in our study expressed that at times their practice’s business model placed greater pressure on their time with patients and personal remuneration, and consequently compromised their ability to practise PCC. Providers in the U. K system have experienced the same issue. A lack of time with patients, and reduced ability to deliver PCC has been reported to result in a loss of professional autonomy and diminished job satisfaction for GPs [31]. Responsibility to deliver PCC lies with providers but also the wider general practice team [9]. Continuing the education of PCC among all practice staff, with the use of the Map for PCC in a collaborative quality improvement activity has the potential to promote PCC and alleviate the pressure on individual GPs striving for PCC. The development of tools/tool kits that include our new Map for PCC, can prompt GPs with new ideas to deliver PCC and support GPs to re-hone their skills and knowledge regarding PCC.

A lack of time, financial pressure and the gatekeeper role were noted by GPs in our study as health system factors that contributed to compromising PCC. Participants’ proposed collaboration with other health professionals as a valuable strategy to alleviate GP time pressure and support PCC. This supports a recently published systematic review and qualitative investigation that reported collaborative care initiatives helped to alleviate individual GP workload, prevent GP burnout and support PCC [32]. Our findings add to the literature valuing the collaboration of care with other health professionals and a patient’s social support network as supportive of PCC.

Patient advocates in our focus groups highlighted the opportunity for environmental design, general practice culture and reception staff to facilitate PCC. In wider research, effective health care space design has been reported to reduce stress, anxiety, and increase patient satisfaction [33]. In hospitals, environmental characteristics (e.g. cleanliness of the space) have been reported to positively influence patient perceptions of patient-centeredness [34]. Of note, providers in our study did not raise consideration of the patient-centredness of their practice environment as an issue. This concept was emphasised in the map for PCC as a practical consideration for GPs to implement PCC and ensure patients feel safe and at ease when they engage with general practice. A patient-centred environment was outlined as supportive of all other aspects of the model, such as forming relationships with providers and involvement in care. Future research should explore novel interventions to assess and optimise the general practice environment to be conducive to and support PCC in practice.

### Strengths and limitations

A strength of this study is that it was based on consultation with the users and beneficiaries of PCC. However, nearly all participants were based in an Australian, urban general practice setting. The applicability of the new Map for PCC outside this context is unknown. Qualitative description presented data in a language used by participants, which supported the new Map for PCC to be credibly contextualised to the ‘real world’ [35]. This study made explicit that change in practice is required to support the effective, universal delivery of PCC in accordance to our advanced model. This is important because innovation theory indicates that providers are more likely to implement practice change if there is evidence that change is required [36].

The synthesis of patient advocate voices with GP voices was essential to informing a model that captures the ‘real world’ understanding of PCC. Patient advocate voices are informed by their training, prior experiences with their GP and own health care, and their knowledge of the health care system. While we did not collect detailed information on the characteristics of patient advocates, their views are likely to be unrepresentative of their patient peers and this may introduce bias to our model. However, patient advocates have demonstrated that their understanding of PCC is highly individual and is grounded in their personal experience [21]. Patient advocates were essential to the development of our PCC model because of their likely deeper understanding of the health system and informed perspectives of health care compared with typical patients.

## Conclusions

This study has advanced our conceptual understanding of PCC in the ‘real world’. *Putting Patients First: A Map for PCC* is a valuable tool for patients, providers and health systems that needs to be embedded into tools or support kits for PCC*.* A novel finding of this study is the importance of the general practice environment, and all staff within the environment for PCC delivery. The physical environment and the role of all general practice staff needs to be a focal point in any analyses or initiatives in the pursuit of PCC.

## Supplementary Information

**Additional file 1: Supplementary Table 1**. Participant Suggestions to Improve the Model.

## Data Availability

The datasets used and/or analysed during the current study are available from the corresponding author on reasonable request.

## Abbreviations

ARIA: Accessibility/remoteness index of Australia
PCC: Patient-centred care
GP: General practitioner
PA: Patient advocate
