# Supplementary material for: Putting patients first: development of a patient advocate and general practitioner-informed model of patient-centred care
Source: BMC Health Serv Res. 2021 Mar 20;21:261. doi: 10.1186/s12913-021-06273-y (PMC7981870; doi:10.1186/s12913-021-06273-y)
Supplement: Supplementary file 1 — Additional file 1: Supplementary Table 1. Participant Suggestions to Improve the Model. [file 12913_2021_6273_MOESM1_ESM.docx]

*Supplementary Table 1. Participant Suggestions to Improve the Model*

| **Message** | **Illustrative Quotations** |
| --- | --- |
| Change language: “halfway” | GP7: In the finding common ground, meeting halfway would suggest that clinicians…  GP4: Half wrong  GP7: The other extreme. So, it’s just using skills to build trust and form partnerships.  GP4: Leave out meet halfway  GP7: It suggests that they’re actually adversarial at the start. |
| The model is lacking medical details | PA6: The first one [component] doesn’t seem to have anything medical in it. If i’m going to a clinician I’d really like him to be up to date with my health record, and my health status. |
| The model is lacking beliefs | PA6: And I’d like to just add in there ‘beliefs’. |
| The model is lacking patient and clinician expectations | GP3: And I agree that we have to manage a balance of not only the patient expectation but the clinician expectation too. I mean you can write 10 pages on this, I mean that’s part of what we have to do, balance our expectations towards the patients. And sometimes if that’s not perfect that’s why they’ll go and get another opinion. |
| The model is lacking patient experience | PA7: I think their [patient] experiences should probably be added in there. Our experiential background has so much bearing on our current state of mind and our current wellness. |
| The model is lacking cultural requirements | PA8: Particularly that first one, understanding the values of patients I think is very important, we haven’t touched on that today… their values, their cultural requirements, their beliefs, all of that is so important.  PA12: Where would cultural competency or cultural safety fit?  PA12: I think it should be something like across cultures or whatever, be specific, like I said if we are going to be faithful to the multicultural… |
| The model is lacking a purpose statement | PA12: I think probably for the likes of myself who comes not from a clinical background directly, I would like a purpose defined out there, like a, you know, not a vision, mission and all of that, but a purpose so that whenever someone picks this up or sees it, why, why are we doing this. Maybe a brief, clear and concise statement as a bi-line to the Patient-Centred Care title, that defines what PCC is.  PA12: When all your staff know this is what you are trying to do (because they read the statement), you don’t have to spell out ok pick up the page with the model, you want to instil in your staff and your doctors your ideal, then everyone would work towards it without people telling others how PCC informs every step of the way, every action they take when consulting/treating a patient, including patients with no or low English proficiency. |
| The model is lacking cost for patients | GP11: Cost for patients. I think cost is really important [to PCC]… Because a lot of medications cost money. I really need to see the specialist or whatever. This all costs money… even if you have to refer to the public hospital. It’s the cost of the parking… |
| Lacking health literacy | GP11: Also health literacy! You need to know what they understand, and you need to have that information. |
| Lacking GPs coordinating timely review of their patients | GP12: I try to encourage them [patients] to come and book for follow-up appointments. If there are other minor issues that they want to discuss on the first visit, then they come back to discuss those… I tell them, I think this is not important today, but you can come back and discuss this with me, and we can deal with it dedicatedly for that issue. So, I think to overcome the time limit, I just tell them… unless it’s for acute obviously you manage the most acute condition then triage it or categorise it to less important.  GP11: If they come back for review more frequently you get to know the person a little bit more better. So then you build up that rapport a bit better…  GP11: I think the timeliness of review needed [is important] …you [patients] feel a bit resentful [regarding lengthy wait to for GP review] and therefore the trust relationship isn’t going to be as good. |
